# Predictors of Size for Gestational Age in St. Louis City and County

**DOI:** 10.1155/2014/515827

**Published:** 2014-07-07

**Authors:** Pamela K. Xaverius, Joanne Salas, Candice L. Woolfolk, Frances Leung, Jessica Yuan, Jen Jen Chang

**Affiliations:** Saint Louis University College for Public Health & Social Justice, 3545 Lafayette Avenue, St. Louis, MO 63104, USA

## Abstract

*Objective*. To identify social, behavioral, and physiological risk factors associated with small for gestational age (SGA) by gestational age category in St. Louis City and County. *Methods*. A retrospective cohort study was conducted using birth certificate and fetal death records from 2000 to 2009 (*n* = 142,017). Adjusted associations of risk factors with SGA were explored using bivariate logistic regression. Four separate multivariable logistic regression analyses, stratified by gestational age, were conducted to estimate adjusted odds ratios. *Results*. Preeclampsia and inadequate weight gain contributed significantly to increased odds for SGA across all gestational age categories. The point estimates ranged from a 3.41 increased odds among women with preeclampsia and 1.76 for women with inadequate weight gain at 24–28 weeks' gestational age to 2.19 and 2.11 for full-term infants, respectively. Among full-term infants, smoking (aOR = 2.08), chronic hypertension (aOR = 1.46), and inadequate prenatal care (aOR = 1.25) had the next most robust and significant impact on SGA. *Conclusion*. Preeclampsia and inadequate weight gain are significant risk factors for SGA, regardless of gestational age. Education on the importance of nutrition and adequate weight gain during pregnancy is vital. In this community, disparities in SGA and smoking rates are important considerations for interventions designed to improve birth outcomes.

## 1. Introduction

**Figure 1:**
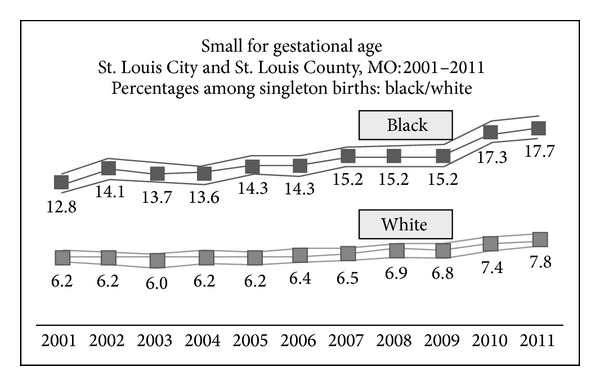
Small for gestational age.

Small for gestational age (SGA) is defined as being less than the 10th percentile for birth weight [1]. Research shows that clinical outcomes are significantly worse for babies that are SGA when compared with those that are normal-for-gestational age [2, 3]. For example, children born SGA have different growth patterns [4], increased risk of neurodevelopmental delays [5, 6], and significantly increased risk of death, compared to babies that are not SGA [7]. In St. Louis City and St. Louis County in 2011, 12.0% of singleton births were SGA, with significant differences based upon race (white = 7.8, 95% confidence interval [CI] = [7.2, 8.3] versus black = 17.7, 95% CI = [16.8 18.7]). Alarmingly, the trend for SGA in this urban area has been significantly increasing since the year 2001 (Mann-Kendall *S* = 48, *P* < 0.05), with SGA among black babies increasing at a significantly faster rate than among white babies [8] (see Figure 1).

Previous epidemiologic studies have examined risk factors for SGA. Ernst and colleagues (2011) found that an elevated biomarker of stress (C-reactive protein) during pregnancy was associated with a 2.9 percent increased odds of SGA [9]. Still other studies have found modifiable and nonmodifiable factors to be significantly associated with SGA including parity, maternal age, maternal smoking, black maternal race, maternal height, maternal prepregnancy weight, maternal education, preeclampsia, weight gain during pregnancy, socioeconomic status, stress, and drug use [10–17]. A prospective cohort study identified several risk factors for SGA among normotensive women, including low maternal birth weight (adjusted odds ratio [aOR] = 1.1, 95% CI = [1.03, 1.14]), smoking (aOR = 1.4, 95% CI = [1.2, 1.6]), low fruit intake before pregnancy (aOR = 1.7, 95% CI = [1.2, 2.6]), and vigorous daily exercise (aOR = 3.2, 95% CI = [1.3, 7.9]) [16]. Thus, some of the risk factors for SGA may be modifiable by changes in behaviors such as diet, smoking, drug use, and stress.

Risks for SGA have also been evaluated across gestational age categories [2, 18]. One study conducted in Sweden examined SGA risk by three gestational age categories: ≤32 (very preterm), 33–36 (moderately preterm), and 36+ (term) weeks' gestation. Preeclampsia and essential hypertension were associated with a significantly increased risk of SGA across each gestational category, but among very preterm infants, the risk for SGA was significantly increased (odds ratio [OR] = 40.5 and OR = 32.5, resp.) [2]. In the same study, smoking was associated with an increased risk of SGA among moderately preterm and term infants [2]. In Missouri, a population based analysis of linked birth certificates from 1989 through 1997 found significant associations between preterm birth (defined as 20–34 weeks' gestational age) and eclampsia, preeclampsia, smoking, and inadequate prenatal care [19]. While risk factors for preterm births have been evaluated with data from Missouri, an understanding of risk factors for SGA by gestational age category might help prevent future morbidity and mortality.

This present study intends to build upon the model presented in the Clausson et al. study by examining risk factors for SGA by gestational age category in St. Louis City and County, a region with rising rates of SGA and large racial disparities. We examined social, behavioral, and chronic disease predictors for SGA, overall and stratified by gestational age.

## 2. Materials and Methods

We conducted a retrospective cohort study using a secondary data analysis of birth certificate data and fetal death records obtained from the Missouri Department of Health and Senior Services. Live birth records were obtained from live birth certificates and fetal death records were obtained from fetal death reports that were filed with the Missouri Department of Health and Senior Services in accordance with state law. In Missouri, the birth certificate and fetal death systems have been in place since 1911 and the data geographically covers both St. Louis City and County. Live, singleton births and fetal deaths of white, non-Hispanic women (*N* = 85,550) and black, non-Hispanic women (*N* = 56,467) from 2000 to 2009 were examined.

Demographic characteristics and pregnancy risk factors were obtained from birth certificates and fetal death reports. These included gestational age (≤28, 29–32, 33–36, and 37+ weeks), race (white, black), maternal age (≤19, 20–34, and ≥35), maternal education (<12, ≥12 years), Medicaid (yes, no), parity (nulliparous [first pregnancy], primiparous [second pregnancy], and multiparous [≥ third pregnancy]). Self-reported pregnancy risk factors included preeclampsia (yes, no), chronic hypertension (yes, no), chronic diabetes (yes, no), smoking status during pregnancy (yes, no), inadequate prenatal care (yes, no), and inadequate weight gain (yes, no). Smoking status during pregnancy was defined as any tobacco use during pregnancy. Inadequate prenatal care was defined by the Missouri Department of Health and Senior Services as fewer than five prenatal visits for pregnancies less than 37 weeks' gestation, fewer than eight visits for pregnancies of 37 weeks' gestation or more, or care beginning after the first four months of pregnancy. Inadequate weight gain was calculated based on body mass index (BMI) category before pregnancy, weight gained during pregnancy, and gestational age. BMI was calculated based on self-reported maternal height and weight using Institute of Medicine (IOM) guidelines [20]. The women were classified as underweight, normal weight, overweight, and obese. Weight gained during pregnancy was also self-reported. Based on IOM guidelines, a weight gain of less than the recommended minimum number of pounds by BMI and gestational age was classified as inadequate weight gain [20].

Gestational age was calculated by the Missouri Department of Health and Senior Services using an algorithm of both clinical estimate and length of pregnancy. The clinical estimate was used to determine gestational age when the calculated length of pregnancy was greater than 44 weeks' gestation and when birth weight and length of pregnancy combinations were deemed implausible, including cases where the length of pregnancy was 24–28 weeks' gestation and the birth weight was greater than 2999 grams, the length of pregnancy was 27–32 weeks' gestation and the birth weight was greater than 3999 grams, or the length of pregnancy was greater than 31 weeks' gestation and the birth weight was greater than 500 grams and less than 1000 grams. For all other cases, the calculated length of pregnancy was used to calculate gestational age.

The outcome of interest, size for gestational age, is a binary indicator for SGA. SGA was defined as birth weights less than 90% of other infants who are born at the same gestational age. The cutoff points for SGA are described elsewhere by Alexander et al. and were used to determine SGA classification, using a United States reference [3].

The final sample size for analysis was 142,017 (live births = 141,579; fetal deaths = 438). Before any restrictions, there were 174,558 data points for live births. After restricting for birth weight of 500 or more grams and to white or black women, there were 159,652 remaining live births. Of these remaining cases, 0.06% were implausible gestational age and birth weight combinations, leaving a sample of 159,547 live births. Also, an additional 5% of cases were excluded due to biologically implausible values for height (<40 and >83.875 inches) and weight (<75 and >350 pounds) and trimming the most extreme 1% of BMI values, observations greater than the 99.5 percentile or less than the 0.5 percentile (<16.5986 and >50.7722), to remove outliers, leaving a sample size of 151,216 [21]. Of this sample, 6.3% were excluded due to missing data on any covariate included in analysis, leaving a final sample size of 141,579 live births. There were 1,366 fetal deaths in St. Louis City and County for the 2000–2009 birth cohort. Live births and fetal deaths at 24 weeks' or above gestation and 500 or more grams were included. The fetal death data were also restricted to black and white women, leaving 646 data points. Approximately 0.6% of these cases were found to have implausible gestational age and birth weight combinations, excluding them from analyses and leaving a sample size of 642. Also, an additional 12% of cases were excluded due to biologically implausible values for height (<40 and >83.875 inches) and weight (<75 and >350 pounds) and trimming the most extreme 1% of BMI values (<16.5986 and >50.7722) leaving a sample size of 561 [21]. Cases with missing values were also excluded (22%) leaving a final sample size of 438 fetal deaths.

Multivariate regressions were conducted on each covariate in two analyses, one of pooled data and one of stratified data. The pooled analysis calculated adjusted odds ratios for each covariate. For the stratified analysis, gestational ages were categorized as follows: 24–28 weeks (extremely preterm), 28–32 weeks (very preterm), 33–36 weeks (preterm), and full-term (37 or more weeks). The stratified analysis included four separate multivariable logistic regression analyses for each gestational age category. Covariates used as adjustments in each of the two analyses included maternal race, maternal age, maternal education, Medicaid, prenatal care, weight gain, parity, smoking, diabetes, chronic hypertension, and preeclampsia. The multivariable logistic regression analyses were used to estimate adjusted odds ratios and 95% confidence intervals for risk factors, using SGA as the outcome. All tests were two-tailed at a 0.05 significance level. SAS version 9.2 was used to perform all analyses.

## 3. Results

### 3.1. Pooled Analysis

Among the 142,017 live births and fetal deaths, 14,860 were SGA. Of the SGA infants, 114 (0.77%) were 28 weeks' gestation or less, 374 (2.52%) were 29–32 weeks', 2228 (14.99%) were 33–36 weeks', and 12144 (81.72%) were 37 weeks' gestation or greater. Descriptive statistics and adjusted odds ratios are summarized in Table 1. In reference with term infants, SGA was significantly less likely at 24–28 weeks' gestation (adjusted odds ratio [aOR] = 0.51, 95% CI = [0.65, 0.95], *P* < 0.0001) and more likely to occur in infants born 33–36 weeks' gestation (aOR = 1.07, 95% CI = [1.02, 1.13], *P* < 0.01) compared to 37+ weeks' gestation. SGA was significantly more likely to occur in mothers 35 years of age or older (aOR = 1.09, 95% CI = [1.02, 1.13], *P* < 0.01), compared to mothers 20–34 years of age. Black mothers were significantly more likely to have a SGA infant (aOR = 1.96, 95% CI = [1.88, 2.04], *P* < 0.0001). Medicaid (aOR = 1.13, 95% CI = [1.08, 1.18], *P* < 0.0001), inadequate prenatal care (aOR = 1.16, 95% CI = [1.10, 1.23], *P* < 0.0001), inadequate weight gain (aOR = 1.99, 95% CI = [1.91, 2.08], *P* < 0.0001), nulliparity (aOR = 1.39, 95% CI = [1.33, 1.45], *P* < 0.0001), smoking (aOR = 1.92, 95% CI = [1.83, 2.02], *P* < 0.0001), chronic hypertension (aOR = 1.38, 95% CI = [1.33, 1.45], *P* < 0.0001), and preeclampsia (aOR = 2.43, 95% CI = [2.29, 2.58], *P* < 0.0001) were also more likely to occur among SGA infants. SGA was significantly less likely to occur among multiparous mothers (aOR = 0.94, 95% CI = [0.90, 0.99], *P* < 0.05) and mothers with diabetes (aOR = 0.69, 95% CI = [0.62, 0.76], *P* < 0.0001). Adjusted odds ratios, stratified by gestational age, are summarized in Table 2.

### 3.2. 24–28 Weeks' Gestation

In the first gestational age category, mothers 19 years of age or younger had a 59% decreased odds of SGA, compared to mothers 20–34 years of age (aOR = 0.41, 95% CI = [0.20, 0.85], *P* < 0.05). Women with inadequate weight gain had a 76% increased odds of SGA, compared to women with adequate weight gain (aOR = 1.76, 95% CI = [1.10, 2.80], *P* < 0.05). Women with preeclampsia had 3.41 times the odds of SGA compared to women without preeclampsia (aOR = 3.41, 95% CI = [2.11, 5.52], *P* < 0.0001).

### 3.3. 29–32 Weeks' Gestation

In the second gestational age category, women who were 35 years of age or older had a 42% increased odds of SGA, compared to women 20–34 years of age (aOR = 1.42, 95% CI = [1.05, 1.93], *P* < 0.05). Women with inadequate weight gain had a 74% increased odds of SGA (aOR = 1.74, 95% CI = [1.29, 2.36], *P* < 0.001). Compared to primiparous women, women who were nulliparous had a 39% increased odds of SGA (aOR = 1.39, 95% CI = [1.06, 1.83], *P* < 0.05). Women who were multiparous had a 40% decreased odds of SGA (aOR = 0.60, 95% CI = [0.44, 0.82], *P* < 0.01). Women with chronic hypertension had a 69% increased odds of SGA (aOR = 1.69, 95% CI = [1.03, 2.76], *P* < 0.05). Women with preeclampsia were 2.10 times as likely as women without preeclampsia to have a SGA infant (aOR = 2.10, 95% CI = [1.62, 2.72], *P* < 0.0001).

### 3.4. 33–36 Weeks' Gestation

In the third gestational age category, black women had a 23% increased odds of having a SGA infant, compared to white women (aOR = 1.23, 95% CI = [1.10, 1.37], *P* < 0.001). Women with less than 12 years of education had a 28% decreased odds of SGA (aOR = 0.72, 95% CI = [0.63, 0.83], *P* < 0.0001). Women on Medicaid had an 11% decreased odds of SGA (aOR = 0.89, 95% CI = [0.79, 0.99], *P* < 0.05). Women with inadequate prenatal care had a 16% decreased odds of SGA (aOR = 0.84, 95% CI = [0.72, 0.98], *P* < 0.05). Inadequate weight gain during pregnancy was associated with a 60% increased odds of SGA (aOR = 1.60, 95% CI = [1.41, 1.81], *P* < 0.0001). Compared to primiparous women, nulliparous women had a 28% increased odds of SGA (aOR = 1.28, 95% CI = [1.14, 1.44], *P* < 0.0001). Women who smoked had a 60% increased odds of SGA (aOR = 1.60, 95% CI = [1.40, 1.82], *P* < 0.0001). Women with diabetes had a 28% decreased odds of SGA (aOR = 0.72, 95% CI = [0.59, 0.89], *P* < 0.01). Compared to women without preeclampsia, women with preeclampsia were 2.82 times as likely to have a SGA infant (aOR = 2.82, 95% CI = [2.52, 3.16], *P* < 0.0001).

### 3.5. ≥37 Weeks' Gestation

In the fourth gestational age category, black mothers were 2.18 times as likely to have a SGA infant (aOR = 2.18, 95% CI = [2.09, 2.29], *P* < 0.0001) as white mothers. Women 35 years of age or older had a 10% increased odds of SGA, compared to women in the 20–34 age group, (aOR = 1.10, 95% CI = [1.03, 1.17], *P* < 0.01). Women with less than 12 years of education had a 14% increased odds of SGA (aOR = 1.14, 95% CI = [1.07, 1.20], *P* < 0.0001) compared to those with 12 years or more of education. Women who received Medicaid had a 17% increased odds of SGA (aOR = 1.17, 95% CI = [1.11, 1.22], *P* < 0.0001). Women who received inadequate prenatal care had a 25% increased odds of SGA (aOR = 1.25, 95% CI = [1.18, 1.32], *P* < 0.0001). Women with inadequate weight gain were 2.11 times as likely to have a SGA infant (aOR = 2.11, 95% CI = [2.01, 2.21], *P* < 0.0001) as women who had adequate weight gain. Nulliparous women were 1.42 times as likely to have a SGA infant (aOR = 1.42, 95% CI = [1.35, 1.49], *P* < 0.0001) as primiparous women. Women who were multiparous had a 6% decreased odds of SGA (aOR = 0.94, 95% CI = [0.89, 0.99], *P* < 0.05). Women who smoked were 2.08 times as likely as nonsmokers to have a SGA infant (aOR = 2.08, 95% CI = [1.97, 2.20], *P* < 0.0001). Women with diabetes had a 34% decreased odds of SGA (aOR = 0.66, 95% CI = [0.59, 0.74], *P* < 0.0001). Chronic hypertension was associated with a 46% increased odds of SGA (aOR = 1.46, 95% CI = [1.27, 1.68], *P* < 0.0001). Women with preeclampsia were 2.19 times as likely as those without preeclampsia to have a SGA infant (aOR = 2.19, 95% CI = [2.04, 2.36], *P* < 0.0001).

## 4. Discussion

We found that preeclampsia and inadequate weight gain contributed significantly to an increased odds for SGA across all gestational age categories. The significant point estimates ranged from a 3.41 increased odds among women with preeclampsia and 1.76 increased odds for women with inadequate weight gain at the 24–28 weeks' gestational age category to a 2.19 and 2.11 increased odds for full-term infants, respectively. Among full-term infants, smoking (aOR = 2.08), chronic hypertension (aOR = 1.46), and inadequate prenatal care (aOR = 1.25) had the next most robust and significant impact on SGA. According to the World Health Organization (2011), calcium supplementation, low-dose aspirin before 20 weeks' gestation, antihypertensive and magnesium sulfate for women with severe preeclampsia, and induction of labor are strongly recommended to prevent or treat hypertensive disorders during pregnancy, which includes preeclampsia [22]. In terms of weight gain during pregnancy, guidelines for weight gain were updated by the Institute of Medicine in 2009, but only a small proportion of pregnant women report that their providers talk to them about weight gain during pregnancy [23]. Education on maternal nutrition and the importance of adequate weight gain during pregnancy may be beneficial in this population [24]. Finally, in another paper currently being written and using this same data set, women who received inadequate prenatal care were found to have 23% increased odds of smoking (aOR = 1.23, 95% CI = [1.01, 1.49]) [8]. The recommendations for preventing and treating hypertensive disorders, smoking, and weight gain during pregnancy suggest that early entry into prenatal care may play an important role in ameliorating the growing and disparate prevalence of SGA in this large urban community.

Interestingly, a number of risk factors were found to have a different effect on SGA between the pooled analysis and the stratified analysis. For example, in the pooled analysis, younger age was not significantly associated with SGA; however, in the stratified analysis, younger age had a 69% decreased odds of SGA at the 24–28 week gestational age category. Inadequate prenatal care in the pooled analysis had a 16% increased odds of SGA, but in the stratified analysis, it was found to have a 16% decreased odds of SGA at the 33–36 gestational age category. We suspect that the relationship between risk factors and SGA was obfuscated in the pooled data, and the stratified analysis suggests that earlier entry into prenatal care may be exceptionally important. For example, if younger women delay entry to prenatal care, they may have missed opportunities for clinical interventions at earlier gestational categories. The impact of clinical interventions as it relates to SGA warrants further study.

There were a few notable results that warrant further consideration. In contrast with Clausson et al., for example, we found that women with lower education have significantly decreased odds of SGA at the 33–36 weeks' gestational age (aOR = 0.72). In addition, education and adequate prenatal care were found to have conflicting impacts at 33–36 weeks' gestation in comparison with term (37+ weeks) infants. For example, lower education was found to have significantly reduced odds of SGA at 33–36 weeks (aOR = 0.72) and significantly increased odds for SGA for term infants (aOR = 1.14). Inadequate prenatal care was also found to have significantly decreased odds for SGA at 33–36 weeks (aOR = 0.84) and significantly increased odds for SGA among term infants (aOR = 1.25). Additionally, diabetes was associated with decreased odds of SGA among those 33 weeks' gestation or greater. The risk of macrosomia is greater among women with diabetes; therefore, SGA is less likely to occur within this population [25]. More research is needed to disentangle the relationship between gestational age and these important covariates.

## 5. Strengths and Limitations

This analysis has several limitations. There is no recommended “gold standard” to computing gestational age [26]. The gestational age calculation used for this study was based on the last menstrual period or clinical estimates, and some infants may have been misclassified into the wrong gestational age category [26, 27]. Another limitation to this study has to do with misclassification of SGA, as SGA may be underestimated at earlier gestational ages due to diverse populations and gender differences, and gender information was not available for this analysis [28, 29]. However, when we compared the proportion of SGA in our study with the proportion of SGA in a more recent and diverse sample [29], we found that our proportions differed by less than 3%. This small difference may have clinical implication for care of a SGA infant misdiagnosed as an AGA, but minimal impact when estimating risk among a large population based sample. All covariates were self-reported, increasing the possibility of self-report bias and recall bias, especially regarding tobacco use, prenatal care visits, and weight gain during pregnancy [30, 31]. Although self-reported data introduced bias into the analysis, research has shown that self-reported data provides reasonably accurate data on chronic conditions and can therefore provide useful estimates of the prevalence of certain conditions [32]. In logistic regression, there is a general rule that there should be a minimum of 10 events per predictor variable, a condition that was not met in the 24–28 weeks' gestational age category [33]. Therefore, the results in this category should be interpreted with caution. Residual confounding is also an issue, as with any secondary data analysis, we were limited to variables that are included in the data set, and other important markers of SGA such as stress, intimate partner violence, and drug and alcohol use, for example, were not available. Diabetes was evaluated in the study as well; however, we lacked information on the type of diabetes (I or II). Finally, since the study only included subjects from St. Louis City and County and white and black women, there is decreased external validity, so the results may not be generalizable to other populations. In spite of these limitations, we believe they are outweighed by the strengths of this analysis that include the large population size, which provided enough power to detect differences between groups and the wide range of potential and important confounders that were evaluated.

## 6. Conclusions

Results suggest that gestational age is an important consideration when evaluating risk factors for SGA in St. Louis City and County. Efforts around disparities in SGA and smoking rates are important considerations for any efforts designed to improve birth outcomes in this community. This analysis also reinforces the need for prompt identification and expectant management of women diagnosed with preeclampsia. The impact of preeclampsia on SGA is greatest among those 24–28 weeks' gestation, while the impact of inadequate weight gain on SGA is greatest among term infants (37 weeks' gestation or greater). Women should also be educated on the importance of maternal nutrition and adequate weight gain during pregnancy. Finally, while the pooled analysis showed no significant impact of low education, and slightly lower point estimates regarding Medicaid and inadequate prenatal care, these social determinants were significant predictors of SGA when compared with full-term infants. Thus, interventions that focus on social determinants that promote wellness, such as access to a higher standard of living that might promote high quality education and early access to high quality care, will likely have a dramatic impact not only on the health of pregnant women and their babies, but perhaps on their hopefulness regarding their future.

## Figures and Tables

**Table 1:** Prevalence of factors by small for gestational age, all live births/fetal deaths, St. Louis City and County, 2000–2009 (*N* = 142,017).

Risk/preventive factors	SGA-no	SGA-yes	Adjusted OR (95% CI)
	(*n* = 127,157)	(*n* = 14,860)	
	*n* %	*n* %	
Gestational age (weeks)			
≤28	1289 (1.01%)	114 (0.77%)	0.51 (0.42, 0.63)
29–32	2536 (1.99%)	374 (2.52%)	0.93 (0.83, 1.04)
33–36	15364 (12.08%)	2228 (14.99%)	1.07 (1.02, 1.13)
≥37	107968 (84.91%)	12144 (81.72%)	1.00
Maternal age			
≤19	12801 (10.07%)	2279 (15.34%)	0.97 (0.91, 1.03)
20–34	94866 (74.61%)	10737 (72.25%)	1.00
≥35	19490 (15.33%)	1844 (12.41%)	1.09 (1.03, 1.16)
Maternal education (years)			
<12	17444 (13.72%)	3061 (20.60%)	1.05 (0.99, 1.11)
≥12	109713 (86.28%)	11799 (79.40%)	1.00
Maternal race			
White	79123 (62.22%)	6427 (43.25%)	1.00
Black	48034 (37.78%)	8433 (56.75%)	1.96 (1.88, 2.04)
Medicaid (yes)	47405 (37.28%)	7653 (51.50%)	1.13 (1.08, 1.18)
Medicaid (no)	79752 (62.72%)	7207 (48.50%)	1.00
Inadequate prenatal care (yes)	11958 (9.40%)	2279 (15.34%)	1.16 (1.10, 1.23)
Inadequate prenatal care (no)	115199 (90.60%)	12581 (84.66%)	1.00
Inadequate weight gain	15271 (12.01%)	3180 (21.40%)	1.99 (1.91, 2.08)
Adequate weight gain	111886 (87.99%)	11680 (78.60%)	1.00
Parity			
Nulliparous	50954 (40.07%)	6871 (46.24%)	1.39 (1.33, 1.45)
Primiparous	38990 (30.66%)	3804 (25.60%)	1.00
Multiparous	37213 (29.27%)	4185 (28.16%)	0.94 (0.90, 0.99)
Smoking (yes)	13023 (10.24%)	2742 (18.45%)	1.92 (1.83, 2.02)
Smoking (no)	114134 (89.76%)	12118 (81.55%)	1.00
Diabetes (yes)	4874 (3.83%)	449 (3.02%)	0.69 (0.62, 0.76)
Diabetes (no)	122283 (96.17%)	14411 (96.98%)	1.00
Chronic hypertension (yes)	1946 (1.53%)	373 (2.51%)	1.38 (1.33, 1.45)
Chronic hypertension (no)	125211 (98.47%)	14487 (97.49%)	1.00
Preeclampsia (yes)	6197 (4.87%)	1715 (11.54%)	2.43 (2.29, 2.58)
Preeclampsia (no)	120960 (95.13%)	13145 (88.46)	1.00

**Table 2:** Adjusted odds ratios (aOR) and 95% confidence intervals (CI) for factors associated with small for gestational age, St. Louis City and County, 2000–2009 (*N* = 142,017).

Risk/preventive factors	Strata-gestational age (weeks)							
	Extremely preterm: 24–28 weeks		Very preterm: 29–32 weeks		Late preterm: 33–36 weeks		Full term: 37 + weeks	
	(*n* = 1,403)		(*n* = 2,910)		(*n* = 17,592)		(*n* = 120,112)	
	aOR	95% CI	aOR	95% CI	aOR	95% CI	aOR	95% CI
Maternal race: black	0.78	0.50, 1.22	1.07	0.82, 1.41	1.23	1.10, 1.37	2.18	2.09, 2.29
Maternal race: white	1.00		1.00		1.00		1.00	
Maternal age: ≤19	0.41	0.20, 0.85	0.69	0.47, 1.01	0.93	0.79, 1.10	0.98	0.92, 1.05
Maternal age: 20–34	1.00		1.00		1.00		1.00	
Maternal age: ≥35	0.92	0.50, 1.69	1.42	1.05, 1.93	0.99	0.87, 1.14	1.10	1.03, 1.17
Maternal education: <12	0.95	0.54, 1.67	0.97	0.70, 1.36	0.72	0.63, 0.83	1.14	1.07, 1.20
Maternal education: ≥12	1.00		1.00		1.00		1.00	
Medicaid: yes	1.20	0.77, 1.88	1.05	0.80, 1.39	0.89	0.79, 0.99	1.17	1.11, 1.22
Medicaid: no	1.00		1.00		1.00		1.00	
Inadequate PNC: yes	0.91	0.57, 1.47	0.88	0.64, 1.21	0.84	0.72, 0.98	1.25	1.18, 1.32
Adequate PNC	1.00		1.00		1.00		1.00	
Inadequate weight gain: no	1.76	1.10, 2.80	1.74	1.29, 2.36	1.60	1.41, 1.81	2.11	2.01, 2.21
Adequate weight gain	1.00		1.00		1.00		1.00	
Parity: nulliparous	0.97	0.60, 1.56	1.39	1.06, 1.83	1.28	1.14, 1.44	1.42	1.35, 1.49
Parity: primiparous	1.00		1.00		1.00		1.00	
Parity: multiparous	0.61	0.36, 1.04	0.60	0.44, 0.82	1.06	0.94, 1.19	0.94	0.89, 0.99
Smoking: yes	0.95	0.53, 1.70	1.08	0.77, 1.51	1.60	1.40, 1.82	2.08	1.97, 2.20
Smoking: no	1.00		1.00		1.00		1.00	
Diabetes: yes	1.17	0.43, 3.18	0.78	0.47, 1.31	0.72	0.59, 0.89	0.66	0.59, 0.74
Diabetes: no	1.00		1.00		1.00		1.00	
Chronic hypertension: yes	0.64	0.18, 2.21	1.69	1.03, 2.76	1.12	0.88, 1.44	1.46	1.27, 1.68
Chronic hypertension: no	1.00		1.00		1.00		1.00	
Preeclampsia: yes	3.41	2.11, 5.52	2.10	1.62, 2.72	2.82	2.52, 3.16	2.19	2.04, 2.36
Preeclampsia: no	1.00		1.00			1.00		1.00

## References

[1] World Health Organization (1995). *Physical Status: The Use and Interpretation of Anthropometry: Report of a WHO Expert Committee*.

[2] Clausson B, Cnattingius S, Axelsson O (1998). Preterm and term births of small for gestational age infants: a population-based study of risk factors among nulliparous women. *British Journal of Obstetrics and Gynaecology*.

[3] Alexander GR, Himes JH, Kaufman RB, Mor J, Kogan M (1996). A United States national reference for fetal growth. *Obstetrics & Gynecology*.

[4] Taal HR, Vd Heijden AJ, Steegers EAP, Hofman A, Jaddoe VWV (2013). Small and large size for gestational age at birth, infant growth, and childhood overweight. *Obesity*.

[5] Streimish IG, Ehrenkranz RA, Allred EN (2012). Birth weight- and fetal weight-growth restriction: impact on neurodevelopment. *Early Human Development*.

[6] Moore GS, Kneitel AW, Walker CK, Gilbert WM, Xing G (2012). Autism risk in small- and large-for-gestational-age infants. *The American Journal of Obstetrics and Gynecology*.

[7] Malloy MH (2007). Size for gestational age at birth: impact on risk for sudden infant death and other causes of death, USA 2002. *Archives of Disease in Childhood: Fetal and Neonatal Edition*.

[8] Missouri Information for Community Assessment (2014). *Small for Gestational Age*.

[9] Ernst GDS, De Jonge LL, Hofman A (2011). C-reactive protein levels in early pregnancy, fetal growth patterns, and the risk for neonatal complications: the generation R Study. *American Journal of Obstetrics & Gynecology*.

[10] Salihu HM, Salinas A, August EM, Mogos MF, Weldeselasse H, Whiteman VE (2012). Small size for gestational age and the risk for infant mortality in the subsequent pregnancy. *Annals of Epidemiology*.

[11] Heaman M, Kingston D, Chalmers B, Sauve R, Lee L, Young D (2013). Risk factors for preterm birth and small-for-gestational-age births among Canadian women. *Paediatric and Perinatal Epidemiology*.

[12] Campbell MK, Cartier S, Xie B, Kouniakis G, Huang W, Han V (2012). Determinants of small for gestational age birth at term. *Paediatric and Perinatal Epidemiology*.

[13] Sebayang SK, Dibley MJ, Kelly PJ, Shankar AV, Shankar AH (2012). Determinants of low birthweight, small-for-gestational-age and preterm birth in Lombok, Indonesia: analyses of the birthweight cohort of the SUMMIT trial. *Tropical Medicine and International Health*.

[14] van den Berg G, van Eijsden M, Galindo-Garre F, Vrijkotte TGM, Gemke RJBJ (2013). Smoking overrules many other risk factors for small for gestational age birth in less educated mothers. *Early Human Development*.

[15] Xue F, Willett WC, Rosner BA, Forman MR, Michels KB (2008). Parental characteristics as predictors of birthweight. *Human Reproduction*.

[16] McCowan LME, Roberts CT, Dekker GA (2010). Risk factors for small-for-gestational-age infants by customised birthweight centiles: data from an international prospective cohort study. *BJOG*.

[17] Lang JM, Cohen A, Lieberman E (1992). Risk factors for small-for-gestational-age birth in a preterm population. *The American Journal of Obstetrics and Gynecology*.

[18] Kajantie E, Phillips DIW, Andersson S (2002). Size at birth, gestational age and cortisol secretion in adult life: foetal programming of both hyper- and hypocortisolism?. *Clinical Endocrinology*.

[19] Kistka ZA-F, Palomar L, Lee KA (2007). Racial disparity in the frequency of recurrence of preterm birth. *American Journal of Obstetrics and Gynecology*.

[20] Rasmussen KM, Yaktine AL (2009). *During Pregnancy: Reexamining the Guidelines*.

[21] Centers for Disease Control and Prevention *Biologically Implausible Values*.

[22] World Health Organization (2011). *WHO Recommendations for Prevention and Treatment of Pre-Eclampsia and Eclampsia: Evidence Base*.

[23] McDonald SD, Pullenayegum E, Taylor VH (2011). Despite 2009 guidelines, few women report being counseled correctly about weight gain during pregnancy. *American Journal of Obstetrics and Gynecology*.

[24] Davis RR, Hofferth SL, Shenassa ED (2014). Gestational weight gain and risk of infant death in the United States. *The American Journal of Public Health*.

[25] Šegregur J, Buković D, Milinović D (2009). Fetal macrosomia in pregnant women with gestational diabetes. *Collegium Antropologicum*.

[26] Ananth CV (2007). Menstrual versus clinical estimate of gestational age dating in the United States: temporal trends and variability in indices of perinatal outcomes. *Paediatric and Perinatal Epidemiology*.

[27] Alexander GR, Tompkins ME, Petersen DJ, Hulsey TC, Mor J (1995). Discordance between LMP-based and clinically estimated gestational age: Implications for research, programs, and policy. *Public Health Reports*.

[28] Halileh S, Abu-Rmeileh N, Watt G, Spencer N, Gordon N (2008). Determinants of birthweight; gender based analysis. *Maternal and Child Health Journal*.

[29] Olsen IE, Groveman SA, Lawson ML, Clark RH, Zemel BS (2010). New intrauterine growth curves based on United States data. *Pediatrics*.

[30] Lydon-Rochelle MT, Cárdenas V, Nelson JL, Tomashek KM, Mueller BA, Easterling TR (2005). Validity of maternal and perinatal risk factors reported on fetal death certificates. *The American Journal of Public Health*.

[31] Northam S, Knapp TR (2006). The reliability and validity of birth certificates. *JOGNN—Journal of Obstetric, Gynecologic, and Neonatal Nursing*.

[32] Martin LM, Leff M, Calonge N, Garrett C, Nelson DE (2000). Validation of self-reported chronic conditions and health services in a managed care population. *American Journal of Preventive Medicine*.

[33] Vittinghoff E, McCulloch CE (2007). Relaxing the rule of ten events per variable in logistic and cox regression. *The American Journal of Epidemiology*.

